# Moving beyond annual data reports: A blueprint for communicating and disseminating actionable intelligence

**DOI:** 10.1017/cts.2022.399

**Published:** 2022-05-10

**Authors:** Raquel Ruiz, Ann Schwartz, Elissa Orlando, Deborah Ossip, Martin S. Zand, Ann Dozier

**Affiliations:** 1 University of Rochester Clinical and Translational Science Institute, Rochester, NY, USA; 2 Center for Leading Innovation and Collaboration (CLIC), CTSA Program Coordinating Center, Rochester, NY, USA; 3 Department of Public Health Sciences, University of Rochester, Rochester, NY, USA; 4 Department of Medicine, Division of Nephrology, University of Rochester, Rochester, NY, USA

**Keywords:** Data to Action, translational science, common metrics, evaluation, clinical research, communication, dissemination

## Abstract

Identifying and disseminating actionable intelligence is a challenging task that requires thoughtful planning. The National Center for Advancing Translational Sciences instituted the Common Metrics Initiative with the goal of evaluating the Clinical and Translational Science Awards (CTSA) Programs using a standard set of metrics. Initially managed by Tufts University, the Center for Leading Innovation and Collaboration (CLIC) at the University of Rochester began leading this initiative in 2017. In directing this work, CLIC created a framework for communicating and disseminating data insights. *Insights to Inspire* emerged from the need to share strategies and lessons learned to improve metric performance at the local level to a network of 60+ academic research institutions. *Insights to Inspire* employs a mixed methods approach for translating data into actionable intelligence. A series of blogs, webinars, and webcasts were designed to communicate metric-specific strategies used by individual sites to the broader CTSA consortium. A dissemination plan to expand the reach beyond metric stakeholders utilized focused communications including social media channels, network newsletters, and presentations at national meetings. This framework serves as a blueprint for other national evaluation programs interested in a systematic approach to using data insights for continuous improvement.

## Introduction

Data to action, used in public health, business, and other fields, is an existing method to drive decisions through the use of data, most commonly quantitative data. Zakocs *et al.* describe their Data-to-Action Framework, a process that guides practitioners through rapid feedback cycles to generate actionable data to improve implementation of ongoing programs [1]. The importance of actions informed by well-organized and presented data has been emphasized in the literature for business [2,3] and other fields. Similarly, the term “actionable intelligence” has emerged as a call to action to implement new ideas and processes [4] and thus is associated with a tactical plan for use of the data collected [5]. In general, the data-to-action and actionable intelligence literature centers on the use of quantitative data. What is lacking is how *qualitative* data can be used to inform an actionable intelligence process.

To address this gap, and guided by a data-to-action approach, the *Insights to Inspire (I2I)* framework was designed and applied to use both qualitative and quantitative data generated from a multi-site national infrastructure initiative, the Clinical Translational Science Award (CTSA) Program. Specifically, the impetus for this process stemmed from requests from participating institutions to learn how others were managing challenges, to gather fresh ideas to improve/enhance their performance on program metrics, and to make the metrics locally usable.

The Common Metrics Initiative (CMI) was designed to assess and optimize the CTSA Program’s overall impact on the nation’s health [6]. Establishing a set of standard evaluation measures across the funded CTSA Program institutions helped focus program activities, streamlined data collection, and demonstrated measurable progress toward program goals, including improvements in research translation and workforce development [7]. The initial implementation of the CMI began in 2015 by Tufts University and is fully described [8]. In 2017, the Center for Leading Innovation and Collaboration (CLIC) assumed responsibility for the continued development and implementation of new metrics (Informatics Metric), as well as improvement and maintenance of the three original metrics: Median IRB Review Duration, Careers in Clinical & Translational Research, and Pilot Funding and Publications. Tufts University group was charged with establishing the initiative’s infrastructure and initial implementation; CLIC focused on engaging participating institutions by making the initiative useful, meaningful, and locally actionable to their work.

In this report, we describe analysis of the CMI qualitative data across four years of results to identify commonalities within each metric and to then share those insights across the CTSA network. We highlight how moving beyond a focus on individual institutions demonstrates the power of aggregating and translating multi-institution qualitative data into actionable intelligence. The application of qualitative analyses to create actionable data-based narratives is defined. Finally, we discuss how the resulting *I2I* framework in the CTSA consortium is also applicable to other national evaluation programs and networks.

### Beyond the Usual Reporting Practices

The flow of reports summarizing aggregate data is commonly unidirectional, from the report developer to the stakeholder. This places the impetus on the stakeholders to use the report in a manner that is most meaningful to them. CLIC’s framework represented a change in both approach and perspective; we used a structured approach for collecting actionable intelligence using the quantitative metric values and qualitative institution narratives. Coupled with focused interviews, these elements provided CLIC with content that could be shared across the CTSA network. Over three annual iterations, the framework was implemented and adapted to address changes in the metrics collected and the strategic direction of the CTSA Program.

CLIC’s primary goal was to provide an annual CMI report to the stakeholders – the 60+ CTSA Program institutions and NCATS. This annual report represented a major step forward in the maturation of the network-wide evaluation initiative; it was the first time that CTSA consortium data had been aggregated and reported back to stakeholders. The report enabled institutions to see how their own metric values compared to de-identified data from their peers across the network. Following the distribution of the first annual report, stakeholder feedback indicated a strong interest in improving institution metric performance by connecting with other CTSA institutions experiencing similar challenges and learning from them by sharing strategies.

**Fig. 1:**
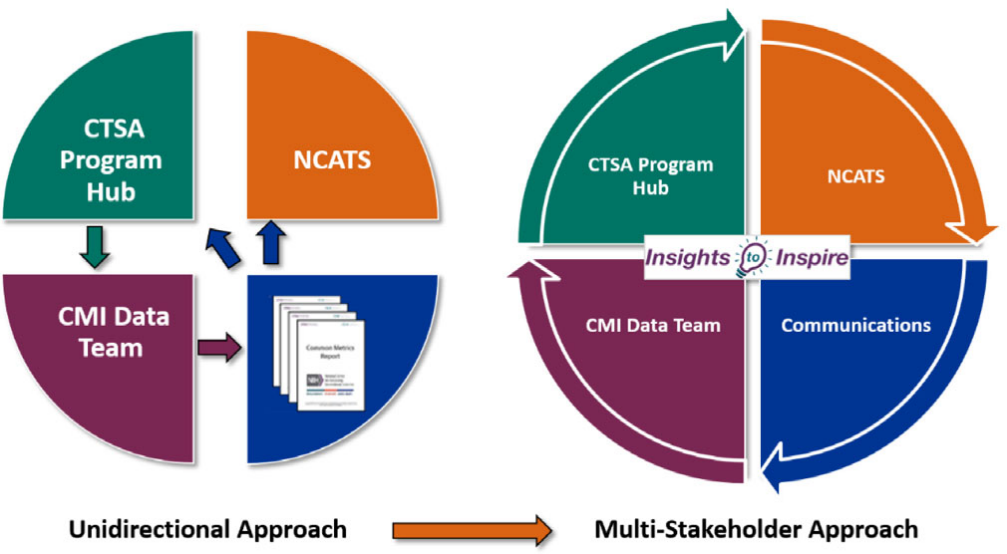
Translating data into action (CTSA – Clinical and Translational Science Awards; NCATS – National Center for Advancing Translational Sciences; CMI – Common Metrics Initiative).

After the first annual report, CLIC created a strategy to move from a unidirectional to a multi-stakeholder framework (see Fig. 1), *Translating Data into Action.* This systematic approach was designed to implement a data-to-action strategy to stimulate conversation, collaboration, and change. Initially, CLIC provided reports to NCATS and the institutions. The launch of *Insights to Inspire* (*I2I*) transformed the reports into an opportunity for stakeholder engagement, communication, and collaboration.

**Fig. 2.**
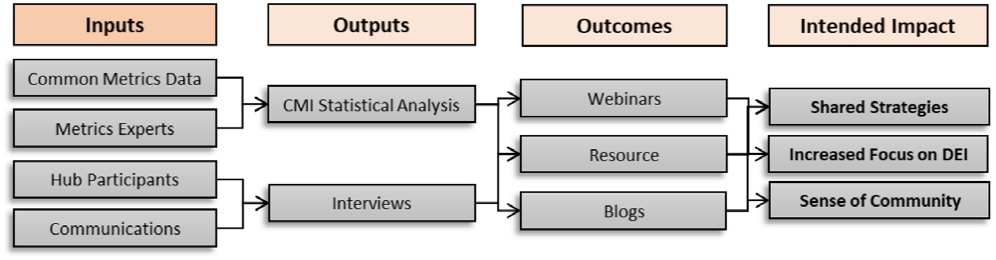
Stakeholder-engaged approach from inputs to intended impact (CMI – Common Metrics Initiative; DEI – Diversity, Equity, and Inclusion).

Fig. 2 below describes the inputs from the consortium which were used for analysis and to inform the interviews. The outcomes outlined resulted from these outputs. The potential impact of the products is the sharing of metric strategies for institutions to reference and to focus on increasing workforce diversity.

## Methods

### Common Metrics Process

CLIC was designated as the “honest broker” for CMI data charged with collecting, collating, cleaning, aggregating, and reporting the data annually. The data were submitted through an online reporting system in which institutions were expected to report both quantitative metric values and supporting qualitative information relative to each value. This framework was implemented with four metrics [9]: Median IRB Review Duration (shorten the time to receive IRB approval), Careers in Clinical & Translational Research (measure the outcomes of two clinical research training programs), Pilot Funding Publications (dissemination of important research study findings), and Informatics (improve data interoperability). Here we describe the method for arriving at the stories starting with the strategic management used for the narratives through selection of the institutions, theme identification, interviews, and finally the communication and dissemination stage.

### Strategic Management Framework

As part of the CMI, the Results-Based Accountability™ (RBA) [10] had been previously selected by NCATS to serve as a strategic management tool to assist institutions in improving their metric values [6]. Institutions entered their locally calculated metric values into an online software platform, which used the data to create a line graph. This graph created a “curve” which enabled institutions to ask, *“Are our data trending in the right direction?”* Institutions prepared Turn-the-Curve (TTC) plans [11] that included four sections: the *Story Behind the Curve* that described what institutions had done to improve the metric value; *Partners* who could help improve the metric; *What Works* that included methods that may be used for improvement; and *Strategies* describing what actions institutions would take in the upcoming year. These sections were used for qualitative analysis and were the basis for the evolution of the multi-stakeholder approach of *Insights to Inspire*.

### Featured Institution Selection

For the qualitative analysis, we identified institutions based on improvement in their metric. Qualitative analyses were intentionally limited to institutions that showed the largest quantitative improvement in their metric data or ranked in the top 50% of a metric. These institutions were designated as “featured institutions.” When there were insufficient year-to-year metric data, as in the case of the Informatics metric, all institutions were included in the qualitative analysis.

### Theme/Story Identification

During year 1, all three original metrics were used for qualitative analysis (IRB, Pilot Funding Publications, and Careers). All four components of institutions’ TTC plans were independently reviewed by two data analysts who read each section and analyzed information based on the RBA framework. Analysts looked for specific information regarding the positive, negative, external, and internal factors in the Story Behind the Curve; Strategies; What Works; and Partners sections.

Year 2 focused on one metric, Careers in Clinical & Translational Research. The analysis focused on strategies employed to diversify the future scientific workforce. This aligned with NCATS’ directive to “…implement education and training programs to provide the required knowledge, skills, and attitudes to aspiring translational scientist at the undergraduate, graduate, early career, and established career levels” [12]. Institutions that showed quantitative improvement in their metric data, or ranked in the top 50% of institutions, were selected for this analysis.

Year 3 included only the newly implemented Informatics metric in its first year of reporting. The Story Behind the Curve sections of all of the institutions’ TTC plans were analyzed using open coding.

### Interview Selection & Process

The goal of this step was to gather qualitative information beyond that reported in the TTC plans through in-depth interviews with the featured institutions. A request for an interview was sent to each institution. In these interviews, which were led by the CMI metric experts and communication teams, questions focused on what activities, processes, people, additional resources were employed or what they perceived as factors for their metric performance improvement.

### Communication

To disseminate *I2I*, a multi-pronged approach was implemented with planned redundancy, including blogs and webinars promoted through the CLIC website to the CTSA Consortium; key stakeholder calls; MailChimp announcements delivered to curated listservs; subscriber-based newsletters; and Twitter campaigns.

## Results

The pairing of metric values and institution narratives resulted in actionable intelligence shared across the network. These results achieved the goals of the *Insights to Inspire* framework – stakeholder engagement, communication, and collaboration.

### Featured Institution Contributors and Actionable Intelligence Collected

**Fig. 3.**
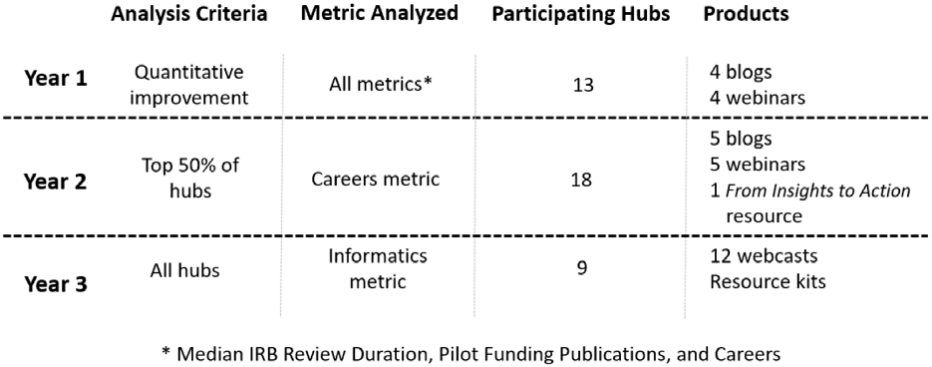
Overview of the insights to inspire approach (IRB – Internal Review Board).

Fig. 3 summarizes criteria, metric(s) analyzed, number of contributors, and products developed.

Year 1: Through the series of blogs disseminated and webinars held over a period of five months, actionable intelligence was shared across the consortium. Thirteen of the seventeen invited institutions participated in this series. Qualitative data analyses resulted in a set of themes that described strategies and tactics used by successful institutions. This actionable intelligence, shared in the first year, included topics ranging from improving IRB turnaround times, selecting appropriate topics for pilot projects, developing recruitment strategies, selecting appropriate scholars, implementing successful mentorship programs, and building peer-to-peer networks. For this first year, all responses from the qualitative analysis were combined and listed in table format in the CMI Multi-Year Data Report: 2015-2017 that was distributed to NCATS and funded institutions.

Year 2: The “Story Behind the Curve” sections of 31 out of 64 unique institutions were selected and open-coded to identify common themes related to programs for both scholars and trainees – which are shown in Table 1. This analysis identified five themes: diversity initiatives, recruitment, application and screening, mentoring, and evaluation and follow-up. These themes were used to develop a resource outlining six strategies for enhancing the diversity of the future scientific workforce, “*From Insights to Action: Enriching the Clinical Research Workforce by Developing Diverse and Inclusive Career Programs.*”

**Table 1.** *I2I* content

Year	Products	Content/Themes
**1: All metrics**	Blogs & Webinars	Using WebCAMP to Track CTSA (Clinical Translational & Science Awards) Pilot Awards Four Strategies for Improving IRB (Internal Review Board) Turnaround Time The Four Stages of a Successful Pilot Funding Publication Program Catapulting the Careers of Future Translational Scientists
**2: Careers metric**	Blogs & Webinars	Making a Commitment to Diversity & Inclusion Building Real Connections: Recruiting Future Clinical Researchers Training Applications: Starting with a Strong Foundation Mentorship: A Bridge to the Future Follow Up and Evaluation: Engagement for Positive Impact
	*From Insight to Action*	Prioritizing Representation Building Partnerships Designing Program Structure Making it Personal Improving through Feedback Winning Endorsement
**3: Informatics metric**	Webcasts	**Overview:** 1: Introduction to Insights to Inspire 2021 2: Language of Informatics 3: Introduction to Informatics 4: Introduction to Maturity Models 5: Importance of Interoperability **Data Quality:** 6: Infrastructure and Data Quality 7: Data Standardization in Data Warehousing **Process Improvements:** 8: Process Improvement **Personnel & Networks:** 9: Partners and Networks 10: Personnel and Interdisciplinary Teams 11: Data Science Education for Informatics **Optimization:** 12: How to Get to Interoperability

Year 3: In response to NIH’s focus on data interoperability, the theme selected for *I2I* Year 3 (2021) was Informatics: The Journey to Interoperability. The categories and content were selected to build a community of expertise among CTSA Programs to create data interoperability across the consortium. Given the recent implementation of this metric and the resulting paucity of narrative content, we determined that Year 3 would require a different approach to develop and share actionable intelligence with the consortium. Instead of narratives from featured institutions, subject matter experts were asked to record a series of webcasts.

Webcast content and potential experts were identified through an in-depth analysis of the previously identified subtopics in the submitted Informatics metric qualitative data. During this process, five broad categories were selected for this series – overview, data quality, process improvements, personnel and networks, and optimization. The first five webcasts served as foundational information and viewers were encouraged to view them in order. The remaining seven webcasts provided more in-depth knowledge of Informatics. The categories are shown in Table 1.

Seven institutions and NCATS were invited to participate in the series, resulting in 12 recorded sessions of 9–28 minutes. Using session transcriptions, CLIC’s marketing personnel identified quotes to inform a Twitter campaign.

### Communication and Dissemination

Annually, CLIC personnel gave presentations during the CTSA Program workgroup meetings to promote the *I2I* program and content, and to highlight the contributions of the featured institutions (N = 27 across the 3 years). The communication staff at each of the 27 featured institutions were notified that their institution was highlighted in a blog or presented on a national webinar. Three of the featured institutions used this information to create their own blogs promoting the accomplishments of their institutions and staff. These blogs were also posted on CLIC’s *Insights to Inspire* webpage. We describe the communication process for each year in detail in the section below.

In Year 1, the initial dissemination of institutions’ successes was conveyed via narratives (TTC plans) highlighting key themes and disseminated through four blog posts on the CLIC website. The four posts (Year 1, 2019) were brief and included key messages to fit the actionable intelligence goal. Citing and associating institutions with the key themes in each blog was an important step in fostering collaboration. Next, these strategies were shared through a series of thematic webinars derived from the blog content. While each blog featured several institutions, two to three institutions were selected to present their strategies, processes, and insights during one of the topic-specific webinars.

For Year 2 (2020), the blog series focused on the importance of diversity and inclusion in the clinical research workforce among women and underrepresented persons. All 18 interviewed institutions of the 33 contacted were included in the five published blogs. Three institutions were selected to present during the accompanying webinars based on their story and ability to convey the narrative. Different from Year 1, the five Year 2 blogs built on each other, starting with the importance of making a commitment to diversity and inclusion followed by recruitment, application and screening, mentoring and training, and follow-up and evaluation. Each blog was associated with a webinar.

In year 3, in addition to the previously used website, the Informatics metric webcasts were posted on both Vimeo and YouTube. The webcasts, PowerPoint slides, and associated transcripts were made available as Resource Kits on the CLIC website [13].

### Participation and Early Outcomes

As of August 31, 2021, CLIC had hosted 10 different topic-specific *I2I* webinars attended by 865 individuals representing 62 unique institutions. In Year 2, follow-up surveys were distributed to webinar attendees (N = 92 respondents from 387 attendees) which indicated that 93% were satisfied or very satisfied with the webinars, 99% indicated that the content was relevant to their role, and 100% indicated that they were somewhat likely or very likely to attend another *I2I* webinar. The survey audience consisted primarily (83%) of institution principal investigators, administrators, evaluators, and education program directors, faculty, and staff.

The participation for each webinar (Years 1 and 2) ranged from 65 to 106 individuals representing 30 to 47 unique institutions (≥45% of CTSA institutions). Webinar attendees during these 2 years included CTSA Program principal investigators, evaluators, administrators, NCATS program directors, and other key stakeholders.

A dedicated *I2I* webpage served as a central location for access to the blogs, recorded webinars, resources, and webcasts. Unique website page views for the blogs ranged from 9 to 27 for Year 1 and increased to a range of 118–418 for Year 2. Since its posting in 2020, there have been 197 unique website page views for *From Insights to Action.* A Twitter campaign began in early 2020 to promote *From Insights to Action* with eight topic-specific tweets to 849 followers. The number of times these tweets were seen (impressions) ranged from 163 to 396. A second Twitter campaign was launched in 2021 to promote Informatics: The Journey to Interoperability. In the first month of posting this content, four tweets received a range of 210–817 impressions per tweet.

## Discussion

In a world driven by data, this framework highlights the value of systematic communication and dissemination strategies to reach a wide variety of stakeholders. The CTSA Program represents a diverse group of stakeholders, including principal investigators, administrators, evaluators, and NCATS personnel, all of whom have a wide range of interests and priorities. These individuals are the decision-makers, strategic thinkers, implementers, and collaborators – the appropriate audience for actionable intelligence generated through our framework.

The CMI is a multifaceted project, involving numerous internal collaborators (communications, website, and administrative logistical support), and external geographically dispersed stakeholders from multiple institutions. It was essential for the internal teams to understand their roles in attaining the goals of the *Insights to Inspire* and at what point(s) in the process they would need to be involved. It required frequent and targeted communications to secure the participation of the institutions. Collaboration with the communications staff/personnel at each institution was integral to the process, in explaining the initiative itself, making the annual metric data results meaningful, and most importantly making the institutional metric results achieved visible and replicable.

The library of content (blogs, webinar presentations, webcasts) [13,14] generated through this process can serve as a foundation for improving metric outcomes: improving the IRB process, increasing diversity and inclusion within our scientific workforce, and increasing the knowledge of institutional staff on informatics. This compendium of strategies, knowledge, and experience provides an opportunity for the network to look beyond their own institutions and explore new ideas. This framework and development of content promotes cross-institution communication and collaboration around shared challenges.

As with many new frameworks, there are areas to be further developed. First, it is difficult to measure the potential reach of the program. *I2I* is a new program, and it takes time to build a regular following of readers. Approximately 42% of the 60+ academic research institutions in the CTSA network contributed to the *Insights to Inspire* program. Not all of the institutions contacted for an interview were available to participate, and for a diverse set of perspectives and lessons learned, it would have been preferable to have full participation. Second, it was not possible to determine whether there was a causal relationship between the strategies listed by the high-performing institutions in their TTC plans and their metric improvement. Last, the impact of Twitter on creating engagement is not quantifiable. The required steps to quantify reach were not taken prior to implementing the campaigns. Twitter activity was sporadic prior to 2021 but regular postings began with the Year 3 campaign. Now that *I2I* is established, the team can be more intentional in measuring the engagement, collaborations fostered, and its potential impact.

As stated earlier, this was a systematic approach designed to implement a data-to-action strategy to stimulate internal and cross-institution conversations, collaborations, and ultimately metric performance improvement. As such, *I2I* served as a method to elicit and communicate strategies to aid institutions in enhancing metric processes. During the initial metric implementation, institutions identified the desire to learn about others’ strategies [8]. The products resulting from the *I2I* begin to address this recommendation identified in the initial implementation process. This process of narrative engagement has provided the space for the institutions to find their own utility and a way of harvesting the wisdom of the consortium.

The metric narrative examination process of *Insights to Inspire* enabled us to highlight the stories and lessons learned specific to a metric or the local context, which includes the program structure and institutional infrastructure. As a result of this process, a community began to organically form centered on the strategies and lessons shared around a particular metric or challenge. Organizing and planning for these “communities of practice” is a potential next step for this approach to bring together different institutions and stakeholders to move the needle on key research-related metrics. A community of practice (CoP) is defined as a group of people who share a common concern, a set of problems, or an interest and who come together to fulfill both individual and group goals [15]. Using a CoP method or similar, institutions can organize around concerns with recruitment and retention of diverse scholars and trainees as an example. This cross-institution collaboration could result in changes of internal processes or policies thus potentially improving the metric quantitative data. The Centers for Disease Control and Prevention has used this CoP framework as a way to strengthen public health [16]. We can begin to learn and assess the impact of the metrics through these communities of practice.

The adaptability and flexibility of this framework for communicating and disseminating actionable intelligence has the potential of replicability to other national evaluation and research networks. These efforts are in alignment with the NIH-NCATS recently released award, PAR-21-293 [17], focused on the use of continuous quality improvement.

## Conclusion

The CMI has matured since its inception in 2015. Its purpose was to fulfill the Institute of Medicine’s report recommendation that a formalized and standardized evaluation process be implemented for individual CTSAs and the CTSA Program [18]. Since 2017, CLIC has grown and expanded the activities of this national initiative in a unique and large research network. One specific way was communicating and disseminating metric-specific strategies with the goal of sharing lessons learned and fostering cross-institution collaboration. *Insights to Inspire* resulted in a blueprint for making data reports more action-oriented. As described here, this framework is flexible and adaptable to the various nuances of a multi-institution metric program and an individual metric.
